# Correction: The influence of freshwater inflow and seascape context on occurrence of juvenile spotted seatrout *Cynoscion nebulosus* across a temperate estuary

**DOI:** 10.1371/journal.pone.0299241

**Published:** 2024-02-15

**Authors:** Shannon D. Whaley, Colin P. Shea, E. Christine Santi, David A. Gandy

The captions for Figs 1–8 are missing from the article. The captions have been provided here:

**Fig 1:**
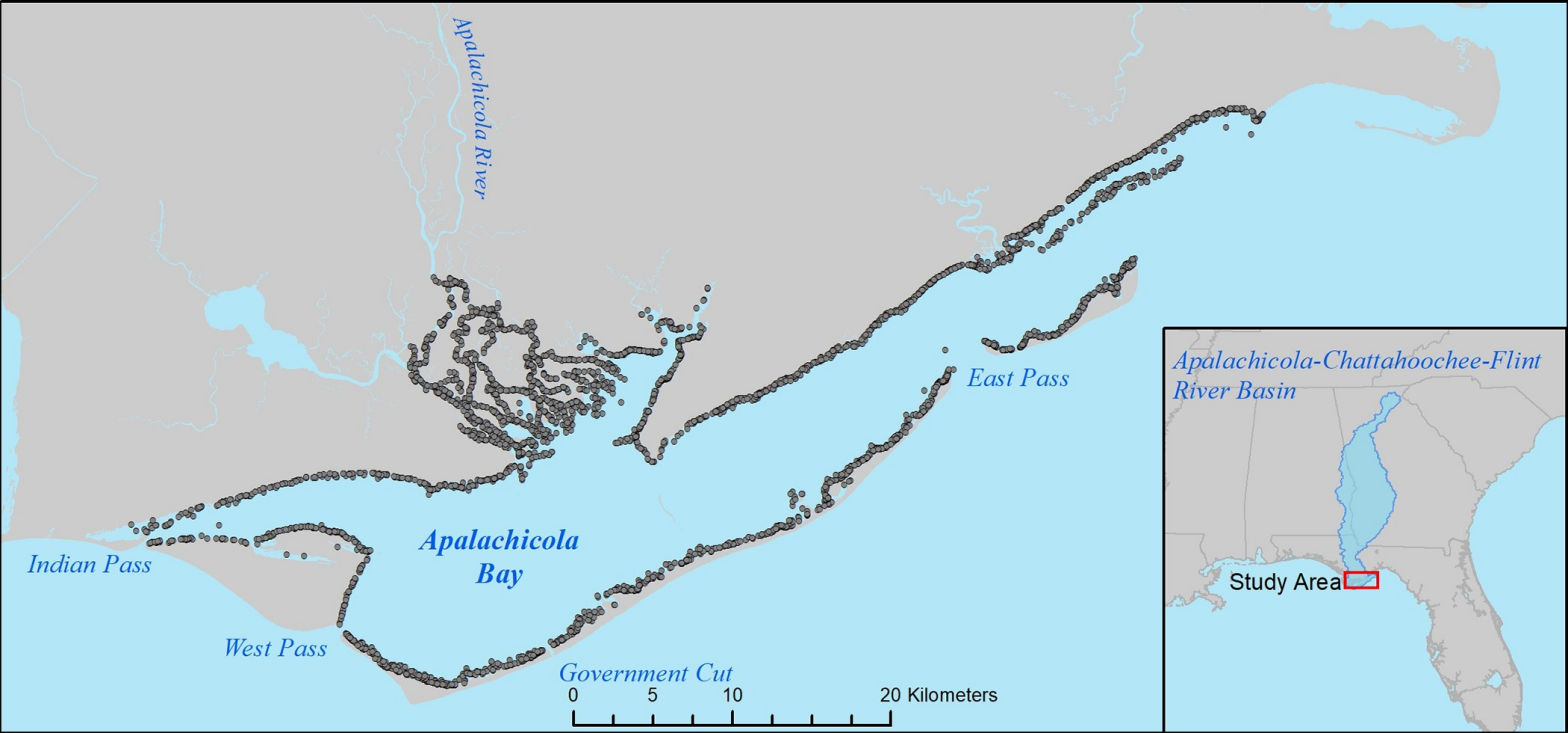
Map of the Apalachicola Bay estuary, Florida, USA. Grey data points indicate fish sampling locations used in this analysis. The Apalachicola River is the main source of freshwater to the estuary. Apalachicola Bay is connected to the Gulf of Mexico through several inlets.

**Fig 2:**
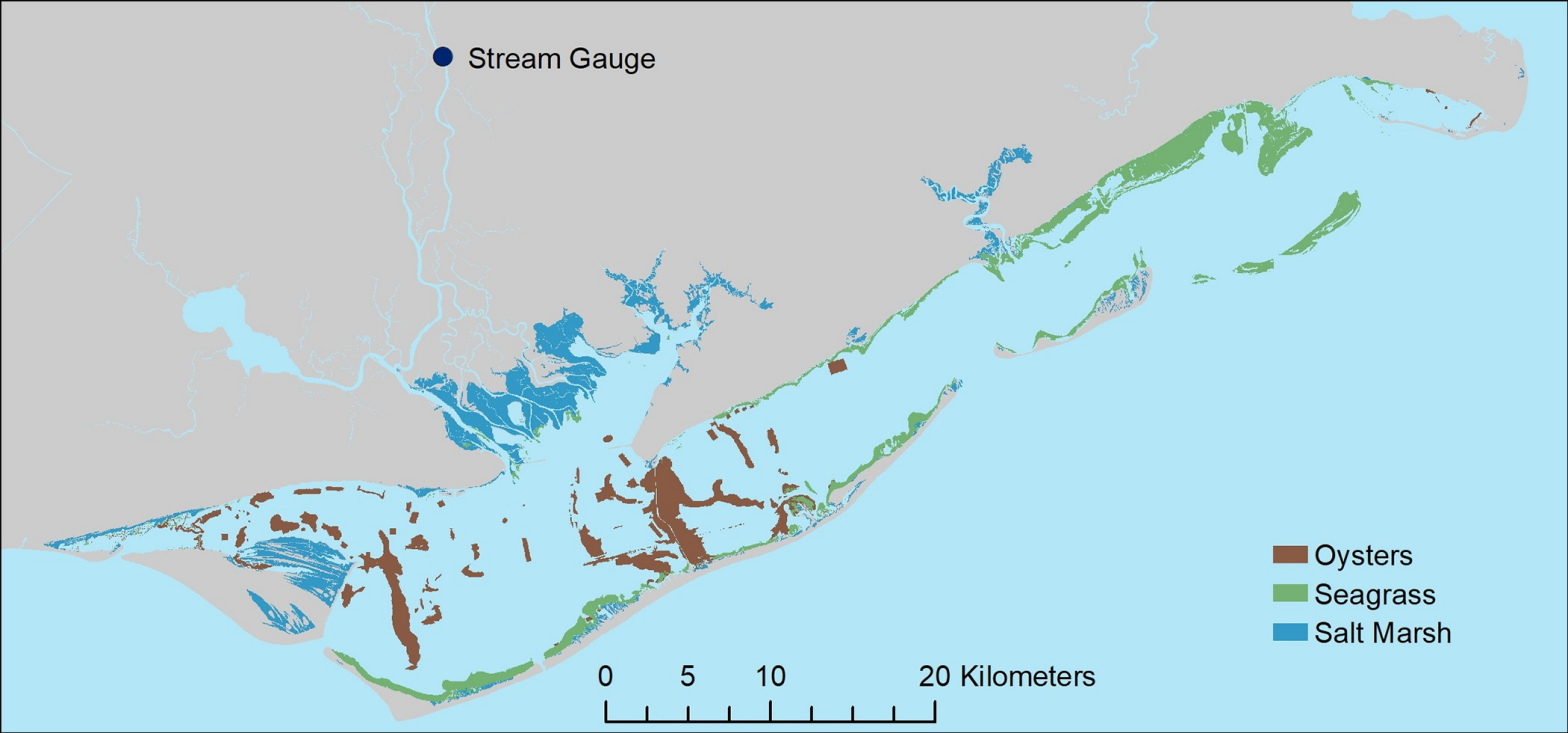
Map of habitats used to develop seascape context metrics. Seagrass beds (green) are distributed in shallow waters primarily along shorelines throughout Apalachicola Bay. Oyster beds (brown) occur primarily in the western half of the Bay; whereas salt marshes (blue) occur mainly along the northern shorelines, particularly in areas near the Apalachicola River. Stream gauge location (blue point) where freshwater flow data was collected for the study.

**Fig 3:**
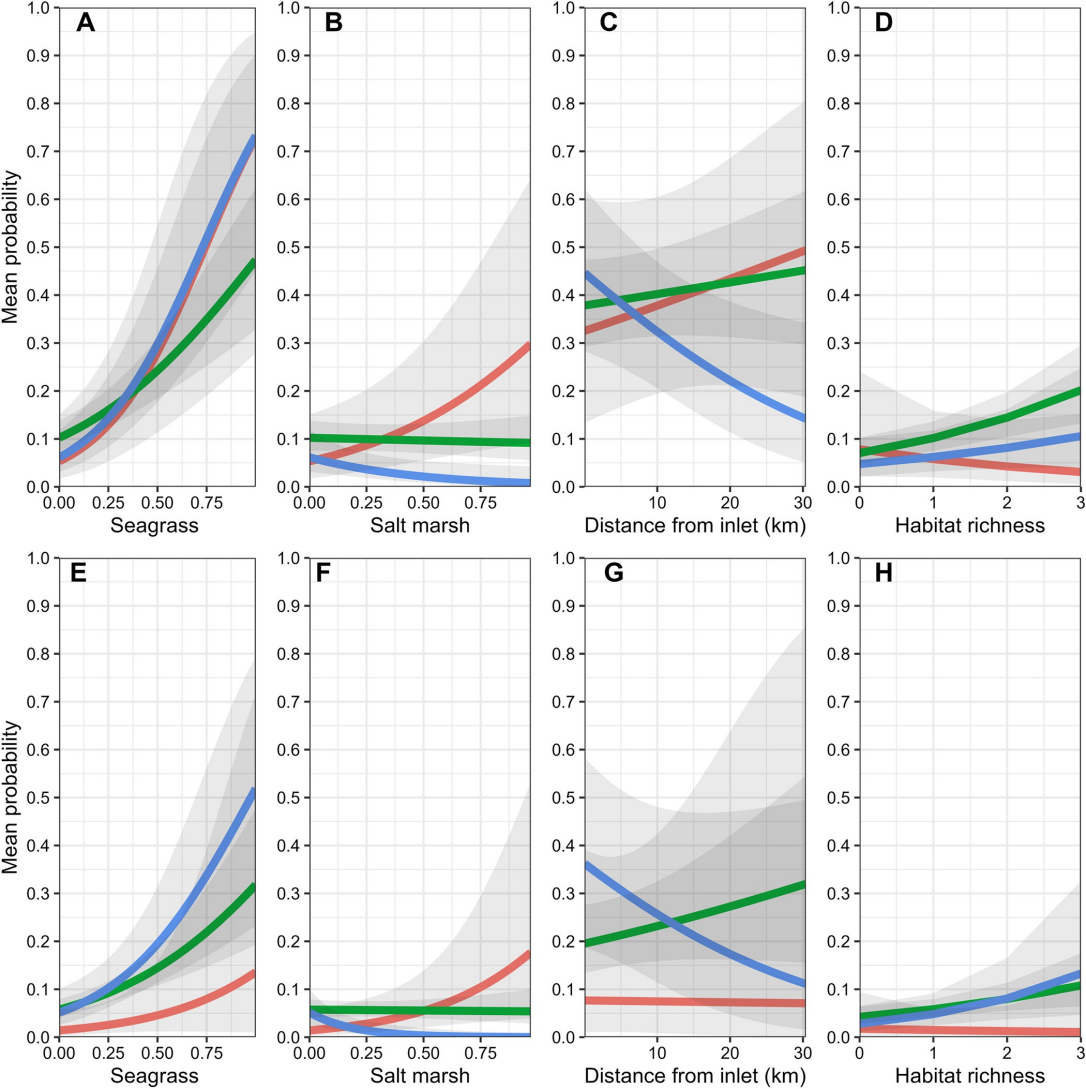
Variable plots for main effects for the 15 – 50mm SL size class (A–D), and the 51 – 100mm SL size class (E–H). Predictions are based on the best-approximating model for each size class using inflow conditions over a 3-month time lag. Red lines indicate dry conditions, blue lines indicate wet conditions, and green lines indicate normal conditions. Shaded regions represent 95% confidence intervals.

**Fig 4:**
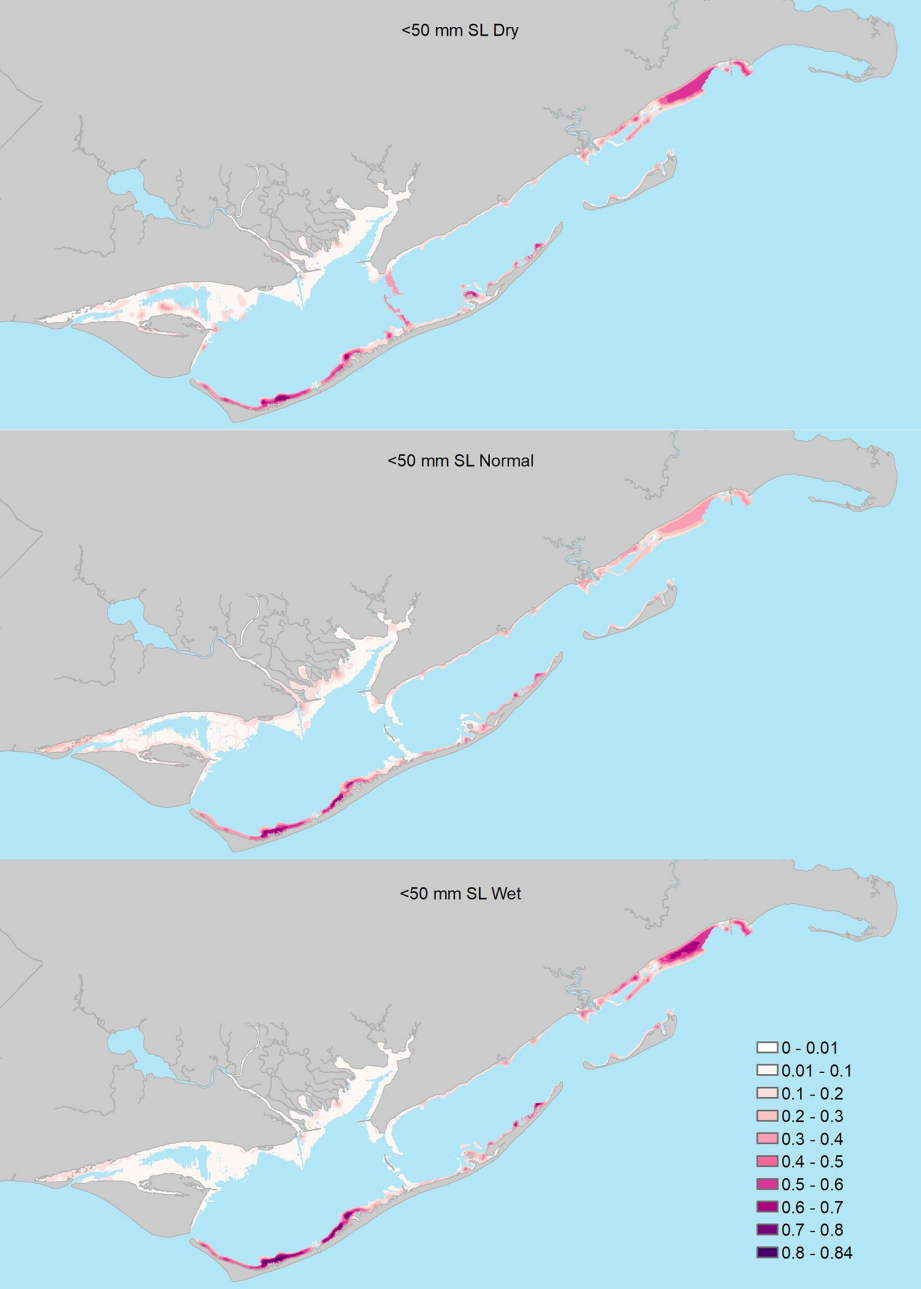
Probability of encountering juvenile seatrout (15 – 50mm Standard Length) in shallow waters of Apalachicola Bay under three freshwater inflow scenarios: A = relatively dry conditions, B = normal inflow, C = relatively wet conditions.

**Fig 5:**
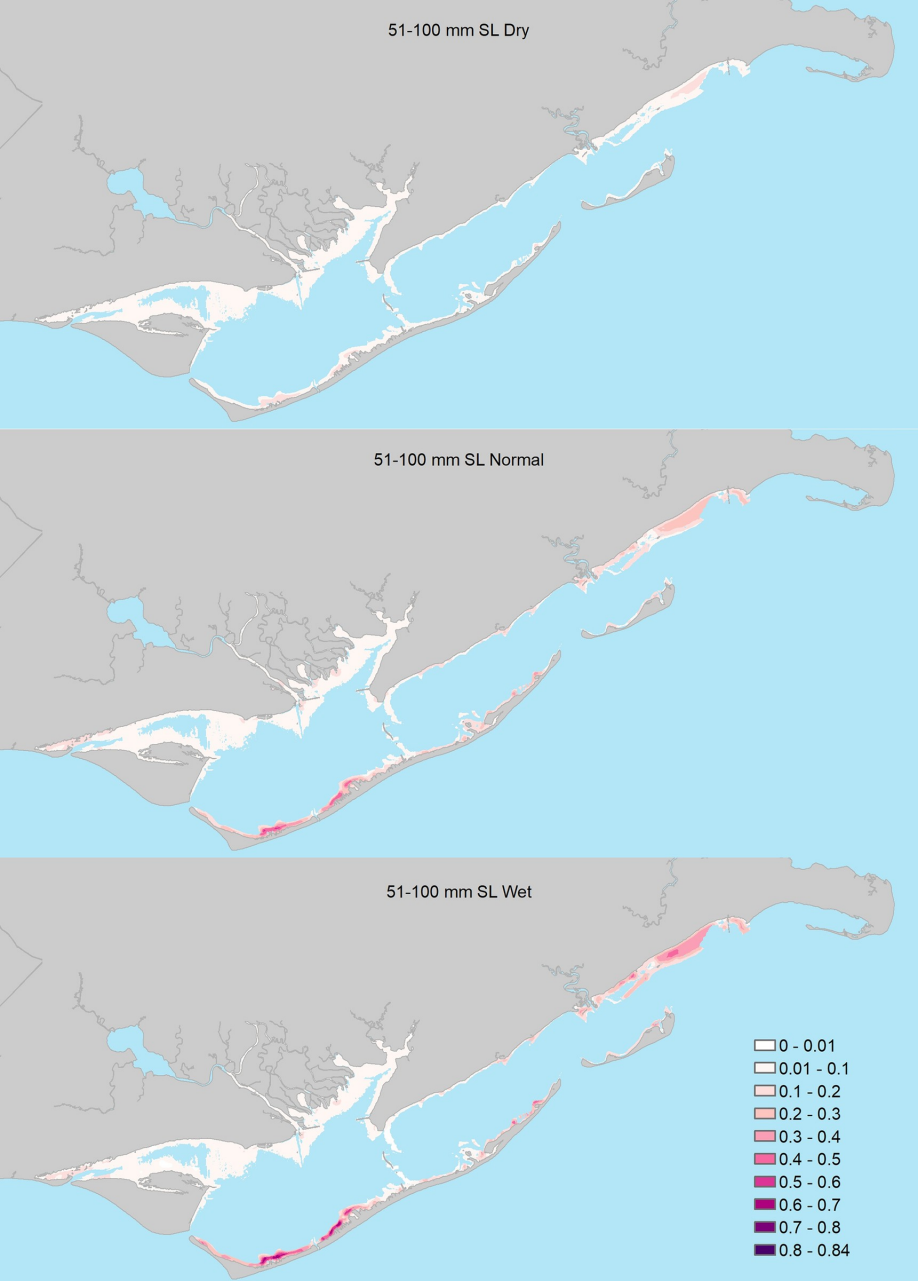
Probability of encountering juvenile seatrout (51 – 100mm Standard Length) in shallow waters of Apalachicola Bay under three freshwater inflow scenarios: A = relatively dry conditions, B = normal inflow, C = relatively wet conditions.

**Fig 6:**
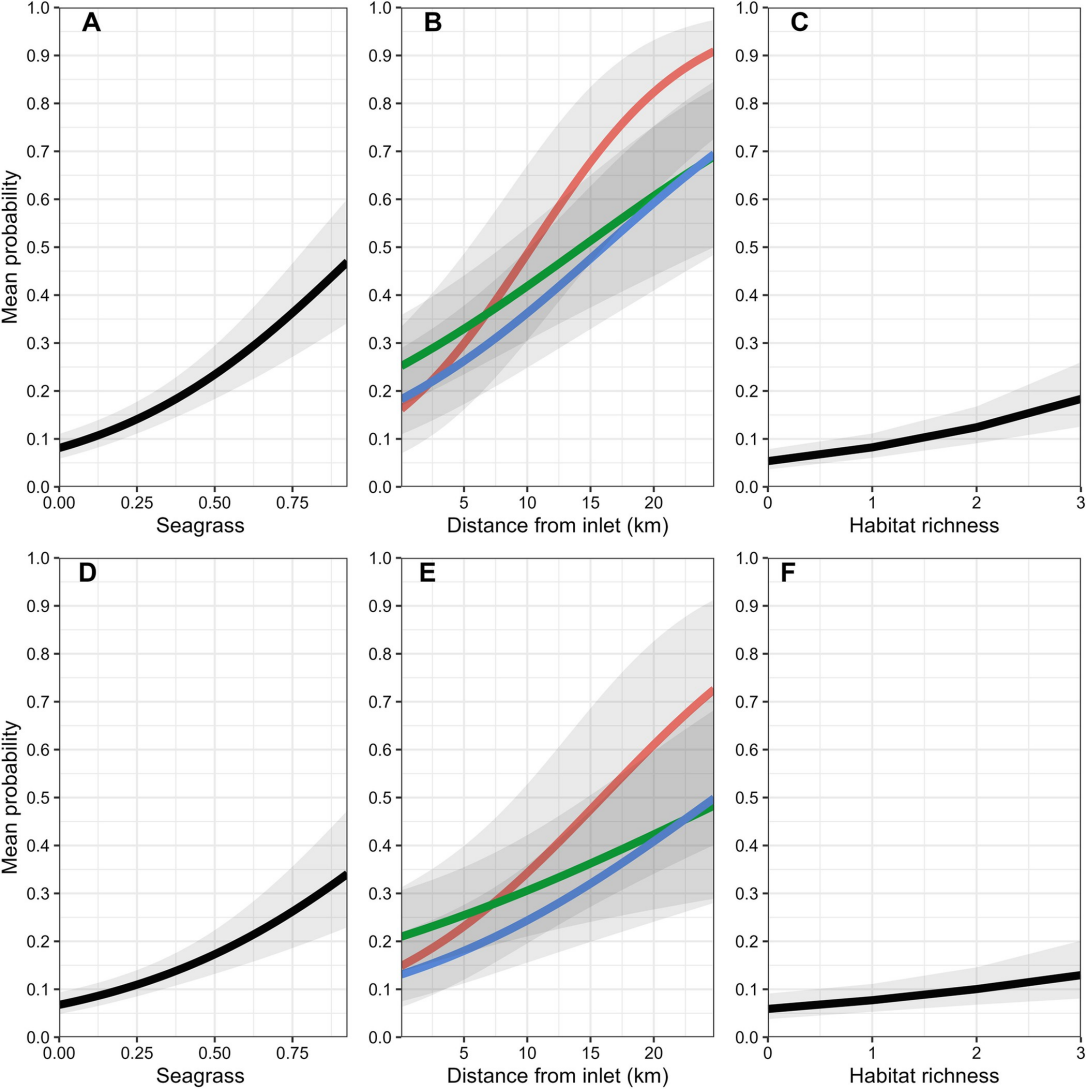
**Variable plots and error bars for main effects for the 101 – 150mm SL size class (A–C), and the 151 – 200mm SL size class (D–F).** Predictions are based on the best-approximating model for each size class using inflow conditions over a 6-month lag. Black lines were averaged across flows. Red lines indicate dry conditions, blue lines indicate wet conditions, and green lines indicate normal conditions. Shaded regions represent 95% confidence intervals.

**Fig 7:**
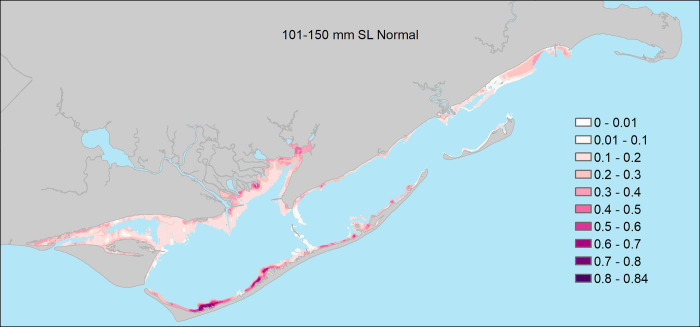
The probability of encountering juvenile seatrout (101 – 150mm Standard Length) in shallow waters of Apalachicola Bay (freshwater inflow was generally unrelated to probability for this size class of seatrout).

**Fig 8:**
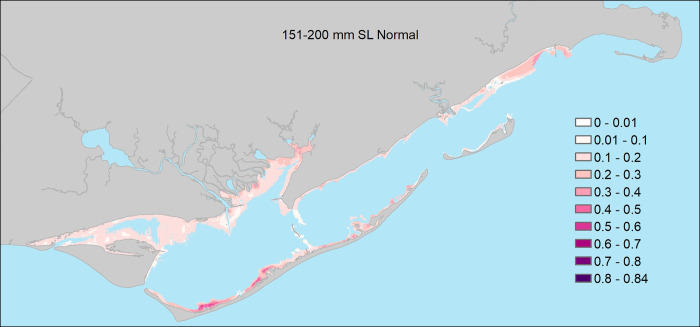
The probability of encountering juvenile seatrout (151 – 200mm Standard Length) in shallow waters of Apalachicola Bay (freshwater inflow was generally unrelated to probability for this size class of seatrout).
