# Supplementary material for: The influence of freshwater inflow and seascape context on occurrence of juvenile spotted seatrout Cynoscion nebulosus across a temperate estuary
Source: PLoS One. 2023 Nov 28;18(11):e0294178. doi: 10.1371/journal.pone.0294178 (PMC10684023; doi:10.1371/journal.pone.0294178)
Supplement: S1 Table — Parameter estimates, standard errors, lower and upper 95% confidence limits, and Wald z-scores (z) and p-values (p) from the confidence set of mixed effects logistic regression models relating seascape-scale, estuary-scale, and hydrologic variables to the probability of encountering ≤ 50 mm spotted seatrout. All values are on the logit (log-odds) scale, random effects are reported as standard deviations, and Imp denotes statistically important relationships based on an alpha level of 0.05. (DOCX) [file pone.0294178.s001.docx]

| **S1 Table.** **Models for ≤50 mm Spotted seatrout.** Parameter estimates, standard errors, lower and upper 95% confidence limits, and Wald z-scores (*z*) and p-values (*p*) from the confidence set of mixed effects logistic regression models relating seascape-scale, estuary-scale, and hydrologic variables to the probability of encountering ≤50 mm spotted seatrout. All values are on the logit (log-odds) scale, random effects are reported as standard deviations, and Imp denotes statistically important relationships based on an alpha level of 0.05. | | | | | | | |
| --- | --- | --- | --- | --- | --- | --- | --- |
| Parameter | Estimate | SE | Lower | Upper | *z* | *p* | Imp |
| *Model 9* |  |  |  |  |  |  |  |
| *Fixed effects* |  |  |  |  |  |  |  |
| Intercept | -2.662 | 0.248 | -3.148 | -2.176 | -10.734 | 0.000 | * |
| NOAA_DEM | 0.066 | 0.079 | -0.089 | 0.222 | 0.838 | 0.402 |  |
| Wet3 | -0.139 | 0.157 | -0.448 | 0.169 | -0.886 | 0.376 |  |
| Dry3 | 0.005 | 0.159 | -0.307 | 0.316 | 0.030 | 0.976 |  |
| CostDistanceInlet | 0.002 | 0.010 | -0.017 | 0.022 | 0.245 | 0.807 |  |
| Seagrass400 | 2.574 | 0.302 | 1.983 | 3.165 | 8.535 | 0.000 | * |
| Saltmarsh400 | -0.362 | 0.277 | -0.906 | 0.182 | -1.305 | 0.192 |  |
| Oysters400 | -1.695 | 0.724 | -3.114 | -0.277 | -2.342 | 0.019 | * |
| HabitatRichness | 0.313 | 0.085 | 0.147 | 0.479 | 3.700 | 0.000 | * |
| Latitude | -0.187 | 0.051 | -0.288 | -0.086 | -3.637 | 0.000 | * |
| Wet3 × Seagrass400 | 0.528 | 0.267 | 0.005 | 1.050 | 1.980 | 0.048 | * |
| Wet3 × Saltmarsh400 | -0.654 | 0.341 | -1.322 | 0.015 | -1.917 | 0.055 |  |
| Wet3 × Oysters400 | 0.758 | 0.633 | -0.483 | 1.999 | 1.197 | 0.231 |  |
| Wet3 × HabitatRichness | -0.036 | 0.075 | -0.183 | 0.112 | -0.475 | 0.635 |  |
| Dry3 × Seagrass400 | 0.392 | 0.270 | -0.137 | 0.921 | 1.452 | 0.147 |  |
| Dry3 × Saltmarsh400 | 0.478 | 0.215 | 0.056 | 0.900 | 2.220 | 0.026 | * |
| Dry3 × Oysters400 | 1.143 | 0.650 | -0.131 | 2.418 | 1.758 | 0.079 |  |
| Dry3 × HabitatRichness | -0.157 | 0.080 | -0.314 | -0.001 | -1.974 | 0.048 | * |
| *Random effect* |  |  |  |  |  |  |  |
| Intercept (Year × Month) | 0.828 |  |  |  |  |  |  |
|  |  |  |  |  |  |  |  |
| *Model 7* |  |  |  |  |  |  |  |
| *Fixed effects* |  |  |  |  |  |  |  |
| Intercept | -2.650 | 0.248 |  |  | -10.670 | 0.000 | * |
| NOAA_DEM | 0.068 | 0.079 |  |  | 0.858 | 0.391 |  |
| Wet3 | 0.012 | 0.246 |  |  | 0.050 | 0.960 |  |
| Dry3 | 0.080 | 0.263 |  |  | 0.303 | 0.762 |  |
| CostDistanceInlet | 0.002 | 0.010 |  |  | 0.161 | 0.872 |  |
| Seagrass400 | 2.559 | 0.302 |  |  | 8.480 | 0.000 | * |
| Saltmarsh400 | -0.349 | 0.277 |  |  | -1.259 | 0.208 |  |
| Oysters400 | -1.717 | 0.725 |  |  | -2.368 | 0.018 | * |
| HabitatRichness | 0.313 | 0.085 |  |  | 3.695 | 0.000 | * |
| Latitude | -0.188 | 0.051 |  |  | -3.645 | 0.000 | * |
| Wet3 × Seagrass400 | 0.390 | 0.317 |  |  | 1.231 | 0.218 |  |
| Wet3 × Saltmarsh400 | -0.599 | 0.347 |  |  | -1.726 | 0.084 |  |
| Wet3 × Oysters400 | 0.817 | 0.634 |  |  | 1.287 | 0.198 |  |
| Wet3 × HabitatRichness | -0.058 | 0.080 |  |  | -0.727 | 0.467 |  |
| Dry3 × Seagrass400 | 0.324 | 0.334 |  |  | 0.970 | 0.332 |  |
| Dry3 × Saltmarsh400 | 0.494 | 0.219 |  |  | 2.255 | 0.024 | * |
| Dry3 × Oysters400 | 1.179 | 0.653 |  |  | 1.805 | 0.071 |  |
| Dry3 × HabitatRichness | -0.169 | 0.085 |  |  | -1.984 | 0.047 | * |
| CostDistanceInlet × Wet3 | -0.008 | 0.011 |  |  | -0.792 | 0.428 |  |
| CostDistanceInlet × Dry3 | -0.004 | 0.011 |  |  | -0.355 | 0.723 |  |
| *Random effect* |  |  |  |  |  |  |  |
| Intercept (Year × Month) |  |  |  |  |  |  |  |
|  |  |  |  |  |  |  |  |
| *Model 8* |  |  |  |  |  |  |  |
| *Fixed effects* |  |  |  |  |  |  |  |
| Intercept | -2.615 | 0.247 | -3.098 | -2.131 | -10.598 | 0.000 | * |
| NOAA_DEM | 0.059 | 0.079 | -0.096 | 0.213 | 0.744 | 0.457 |  |
| Wet3 | 0.089 | 0.132 | -0.169 | 0.347 | 0.675 | 0.500 |  |
| Dry3 | -0.050 | 0.135 | -0.314 | 0.213 | -0.375 | 0.708 |  |
| CostDistanceInlet | -0.002 | 0.010 | -0.022 | 0.018 | -0.173 | 0.863 |  |
| Seagrass400 | 2.515 | 0.299 | 1.929 | 3.100 | 8.422 | 0.000 | * |
| Saltmarsh400 | -0.053 | 0.243 | -0.529 | 0.424 | -0.217 | 0.828 |  |
| Oysters400 | -1.548 | 0.706 | -2.933 | -0.164 | -2.192 | 0.028 | * |
| HabitatRichness | 0.300 | 0.084 | 0.135 | 0.465 | 3.572 | 0.000 | * |
| Latitude | -0.188 | 0.051 | -0.288 | -0.087 | -3.662 | 0.000 | * |
| CostDistanceInlet × Wet3 | -0.020 | 0.008 | -0.035 | -0.004 | -2.533 | 0.011 | * |
| CostDistanceInlet × Dry3 | 0.003 | 0.007 | -0.011 | 0.016 | 0.412 | 0.680 |  |
| *Random effect* |  |  |  |  |  |  |  |
| Intercept (Year × Month) | 0.823 |  |  |  |  |  |  |
|  |  |  |  |  |  |  |  |
| *Model 17* |  |  |  |  |  |  |  |
| *Fixed effects* |  |  |  |  |  |  |  |
| Intercept | -2.631 | 0.247 | -3.115 | -2.146 | -10.637 | 0.000 | * |
| NOAA_DEM | 0.055 | 0.079 | -0.099 | 0.209 | 0.705 | 0.481 |  |
| CostDistanceInlet | 0.000 | 0.010 | -0.019 | 0.020 | 0.050 | 0.960 |  |
| Seagrass400 | 2.521 | 0.299 | 1.936 | 3.107 | 8.439 | 0.000 | * |
| Saltmarsh400 | -0.053 | 0.243 | -0.528 | 0.423 | -0.217 | 0.828 |  |
| Oysters400 | -1.575 | 0.705 | -2.956 | -0.194 | -2.236 | 0.025 | * |
| HabitatRichness | 0.297 | 0.084 | 0.133 | 0.461 | 3.541 | 0.000 | * |
| Latitude | -0.188 | 0.051 | -0.289 | -0.087 | -3.662 | 0.000 | * |
| *Random effect* |  |  |  |  |  |  |  |
| Intercept (Year × Month) | 0.840 |  |  |  |  |  |  |
|  |  |  |  |  |  |  |  |
| *Model 11* |  |  |  |  |  |  |  |
| *Fixed effects* |  |  |  |  |  |  |  |
| Intercept | -2.697 | 0.259 | -3.204 | -2.189 | -10.419 | 0.000 | * |
| NOAA_DEM | 0.060 | 0.079 | -0.095 | 0.214 | 0.758 | 0.448 |  |
| CostDistanceInlet | 0.009 | 0.011 | -0.012 | 0.030 | 0.864 | 0.387 |  |
| Wet1 | 0.434 | 0.315 | -0.184 | 1.051 | 1.375 | 0.169 |  |
| Dry1 | -0.482 | 0.638 | -1.732 | 0.768 | -0.756 | 0.449 |  |
| Seagrass400 | 2.522 | 0.299 | 1.936 | 3.108 | 8.433 | 0.000 | * |
| Saltmarsh400 | -0.051 | 0.243 | -0.527 | 0.425 | -0.210 | 0.834 |  |
| Oysters400 | -1.574 | 0.706 | -2.958 | -0.189 | -2.228 | 0.026 | * |
| HabitatRichness | 0.301 | 0.084 | 0.136 | 0.466 | 3.579 | 0.000 | * |
| Latitude | -0.188 | 0.051 | -0.289 | -0.087 | -3.658 | 0.000 | * |
| CostDistanceInlet × Wet1 | -0.047 | 0.018 | -0.083 | -0.011 | -2.531 | 0.011 | * |
| CostDistanceInlet × Dry1 | 0.007 | 0.036 | -0.064 | 0.077 | 0.183 | 0.855 |  |
| *Random effect* |  |  |  |  |  |  |  |
| Intercept (Year × Month) | 0.829 |  |  |  |  |  |  |
|  |  |  |  |  |  |  |  |
| *Model 5* |  |  |  |  |  |  |  |
| *Fixed effects* |  |  |  |  |  |  |  |
| Intercept | -2.629 | 0.247 | -3.113 | -2.144 | -10.642 | 0.000 | * |
| NOAA_DEM | 0.058 | 0.079 | -0.097 | 0.212 | 0.731 | 0.465 |  |
| CostDistanceInlet | -0.001 | 0.010 | -0.020 | 0.019 | -0.054 | 0.957 |  |
| Wet6 | -0.012 | 0.140 | -0.287 | 0.263 | -0.086 | 0.931 |  |
| Dry6 | -0.163 | 0.142 | -0.442 | 0.116 | -1.146 | 0.252 |  |
| Seagrass400 | 2.516 | 0.299 | 1.930 | 3.102 | 8.419 | 0.000 | * |
| Saltmarsh400 | -0.056 | 0.243 | -0.532 | 0.420 | -0.232 | 0.816 |  |
| Oysters400 | -1.575 | 0.706 | -2.958 | -0.192 | -2.232 | 0.026 | * |
| HabitatRichness | 0.301 | 0.084 | 0.137 | 0.466 | 3.588 | 0.000 | * |
| Latitude | -0.188 | 0.051 | -0.288 | -0.087 | -3.658 | 0.000 | * |
| CostDistanceInlet × Wet6 | -0.014 | 0.008 | -0.031 | 0.002 | -1.743 | 0.081 |  |
| CostDistanceInlet × Dry6 | 0.003 | 0.008 | -0.012 | 0.018 | 0.347 | 0.729 |  |
| *Random effect* |  |  |  |  |  |  |  |
| Intercept (Year × Month) | 0.825 |  |  |  |  |  |  |
|  |  |  |  |  |  |  |  |
| *Model 14* |  |  |  |  |  |  |  |
| *Fixed effects* |  |  |  |  |  |  |  |
| Intercept | -2.628 | 0.247 | -3.112 | -2.145 | -10.657 | 0.000 | * |
| NOAA_DEM | 0.056 | 0.079 | -0.098 | 0.210 | 0.709 | 0.478 |  |
| CostDistanceInlet | 0.000 | 0.010 | -0.019 | 0.020 | 0.038 | 0.970 |  |
| Wet6 | -0.164 | 0.111 | -0.382 | 0.054 | -1.477 | 0.140 |  |
| Dry6 | -0.131 | 0.111 | -0.349 | 0.087 | -1.176 | 0.240 |  |
| Seagrass400 | 2.508 | 0.299 | 1.923 | 3.093 | 8.399 | 0.000 | * |
| Saltmarsh400 | -0.056 | 0.243 | -0.532 | 0.420 | -0.231 | 0.818 |  |
| Oysters400 | -1.580 | 0.705 | -2.963 | -0.197 | -2.240 | 0.025 | * |
| HabitatRichness | 0.299 | 0.084 | 0.135 | 0.464 | 3.565 | 0.000 | * |
| Latitude | -0.187 | 0.051 | -0.288 | -0.087 | -3.648 | 0.000 | * |
| *Random effect* |  |  |  |  |  |  |  |
| Intercept (Year × Month) | 0.8228 |  |  |  |  |  |  |
