# Supplementary material for: The influence of freshwater inflow and seascape context on occurrence of juvenile spotted seatrout Cynoscion nebulosus across a temperate estuary
Source: PLoS One. 2023 Nov 28;18(11):e0294178. doi: 10.1371/journal.pone.0294178 (PMC10684023; doi:10.1371/journal.pone.0294178)
Supplement: S2 Table — Parameter estimates, standard errors, lower and upper 95% confidence limits, and Wald z-scores (z) and p-values (p) from the confidence set of mixed effects logistic regression models relating seascape-scale, estuary-scale, and hydrologic variables to the probability of encountering 51–100 mm spotted seatrout. All values are on the logit (log-odds) scale, random effects are reported as standard deviations, and Imp denotes statistically important relationships based on an alpha level of 0.05. (DOCX) [file pone.0294178.s002.docx]

| **S2 Table.** **Models for 51** – **100 mm Spotted seatrout.** Parameter estimates, standard errors, lower and upper 95% confidence limits, and Wald z-scores (*z*) and p-values (*p*) from the confidence set of mixed effects logistic regression models relating seascape-scale, estuary-scale, and hydrologic variables to the probability of encountering 51 – 100 mm spotted seatrout. All values are on the logit (log-odds) scale, random effects are reported as standard deviations, and Imp denotes statistically important relationships based on an alpha level of 0.05. | | | | | | | |
| --- | --- | --- | --- | --- | --- | --- | --- |
| Parameter | Estimate | SE | Lower | Upper | *z* | *p* | Imp |
| *Model 9* |  |  |  |  |  |  |  |
| *Fixed effects* |  |  |  |  |  |  |  |
| Intercept | -3.360 | 0.292 | -3.933 | -2.787 | -11.494 | 0.000 | * |
| NOAA_DEM | 0.014 | 0.102 | -0.187 | 0.214 | 0.133 | 0.894 |  |
| Wet3 | -0.108 | 0.165 | -0.432 | 0.216 | -0.656 | 0.512 |  |
| Dry3 | -0.203 | 0.205 | -0.605 | 0.200 | -0.986 | 0.324 |  |
| CostDistanceInlet | 0.006 | 0.013 | -0.019 | 0.031 | 0.492 | 0.623 |  |
| Seagrass400 | 2.264 | 0.361 | 1.556 | 2.972 | 6.267 | 0.000 | * |
| Saltmarsh400 | -0.923 | 0.449 | -1.804 | -0.042 | -2.054 | 0.040 | * |
| Oysters400 | -1.419 | 0.987 | -3.354 | 0.515 | -1.438 | 0.150 |  |
| HabitatRichness | 0.336 | 0.105 | 0.130 | 0.542 | 3.192 | 0.001 | * |
| Latitude | -0.138 | 0.061 | -0.257 | -0.019 | -2.266 | 0.023 | * |
| Wet3 × Seagrass400 | 0.304 | 0.303 | -0.290 | 0.897 | 1.003 | 0.316 |  |
| Wet3 × Saltmarsh400 | -1.674 | 0.605 | -2.859 | -0.489 | -2.768 | 0.006 | * |
| Wet3 × Oysters400 | -1.269 | 1.192 | -3.605 | 1.068 | -1.064 | 0.287 |  |
| Wet3 × HabitatRichness | 0.070 | 0.091 | -0.109 | 0.248 | 0.765 | 0.444 |  |
| Dry3 × Seagrass400 | 0.077 | 0.399 | -0.706 | 0.859 | 0.192 | 0.848 |  |
| Dry3 × Saltmarsh400 | 0.617 | 0.299 | 0.031 | 1.203 | 2.065 | 0.039 | * |
| Dry3 × Oysters400 | 0.096 | 1.185 | -2.227 | 2.418 | 0.081 | 0.936 |  |
| Dry3 × HabitatRichness | -0.109 | 0.118 | -0.340 | 0.122 | -0.927 | 0.354 |  |
| *Random effect* |  |  |  |  |  |  |  |
| Intercept (Year × Month) | 0.479 |  |  |  |  |  |  |
|  |  |  |  |  |  |  |  |
| *Model 7* |  |  |  |  |  |  |  |
| *Fixed effects* |  |  |  |  |  |  |  |
| Intercept | -3.351 | 0.297 | -3.933 | -2.769 | -11.281 | 0.000 | * |
| NOAA_DEM | 0.017 | 0.103 | -0.184 | 0.218 | 0.164 | 0.869 |  |
| Wet3 | 0.268 | 0.271 | -0.263 | 0.798 | 0.989 | 0.323 |  |
| Dry3 | -0.104 | 0.371 | -0.831 | 0.624 | -0.280 | 0.780 |  |
| CostDistanceInlet | 0.005 | 0.013 | -0.021 | 0.030 | 0.359 | 0.720 |  |
| Seagrass400 | 2.257 | 0.365 | 1.541 | 2.972 | 6.181 | 0.000 | * |
| Saltmarsh400 | -0.844 | 0.445 | -1.716 | 0.028 | -1.896 | 0.058 |  |
| Oysters400 | -1.448 | 0.988 | -3.385 | 0.490 | -1.464 | 0.143 |  |
| HabitatRichness | 0.334 | 0.105 | 0.128 | 0.540 | 3.172 | 0.002 | * |
| Latitude | -0.140 | 0.061 | -0.260 | -0.020 | -2.290 | 0.022 | * |
| Wet3 × Seagrass400 | -0.019 | 0.351 | -0.706 | 0.668 | -0.054 | 0.957 |  |
| Wet3 × Saltmarsh400 | -1.451 | 0.607 | -2.641 | -0.261 | -2.389 | 0.017 | * |
| Wet3 × Oysters400 | -1.072 | 1.195 | -3.414 | 1.270 | -0.897 | 0.370 |  |
| Wet3 × HabitatRichness | 0.015 | 0.096 | -0.174 | 0.205 | 0.160 | 0.873 |  |
| Dry3 × Seagrass400 | -0.004 | 0.490 | -0.964 | 0.955 | -0.009 | 0.993 |  |
| Dry3 × Saltmarsh400 | 0.643 | 0.305 | 0.046 | 1.241 | 2.111 | 0.035 | * |
| Dry3 × Oysters400 | 0.162 | 1.190 | -2.169 | 2.494 | 0.136 | 0.892 |  |
| Dry3 × HabitatRichness | -0.125 | 0.124 | -0.368 | 0.119 | -1.005 | 0.315 |  |
| CostDistanceInlet × Wet3 | -0.023 | 0.013 | -0.048 | 0.003 | -1.717 | 0.086 |  |
| CostDistanceInlet × Dry3 | -0.005 | 0.017 | -0.038 | 0.028 | -0.317 | 0.751 |  |
| *Random effect* |  |  |  |  |  |  |  |
| Intercept (Year × Month) | 0.480 |  |  |  |  |  |  |
|  |  |  |  |  |  |  |  |
| *Model 8* |  |  |  |  |  |  |  |
| *Fixed effects* |  |  |  |  |  |  |  |
| Intercept | -3.359 | 0.291 | -3.930 | -2.788 | -11.527 | 0.000 | * |
| NOAA_DEM | 0.019 | 0.102 | -0.181 | 0.219 | 0.188 | 0.851 |  |
| Wet3 | 0.249 | 0.126 | 0.002 | 0.496 | 1.979 | 0.048 | * |
| Dry3 | -0.325 | 0.161 | -0.640 | -0.009 | -2.017 | 0.044 | * |
| CostDistanceInlet | 0.002 | 0.013 | -0.023 | 0.027 | 0.174 | 0.862 |  |
| Seagrass400 | 2.319 | 0.352 | 1.629 | 3.010 | 6.584 | 0.000 | * |
| Saltmarsh400 | -0.197 | 0.330 | -0.844 | 0.450 | -0.597 | 0.550 |  |
| Oysters400 | -1.180 | 0.876 | -2.897 | 0.537 | -1.347 | 0.178 |  |
| HabitatRichness | 0.319 | 0.103 | 0.116 | 0.521 | 3.088 | 0.002 | * |
| Latitude | -0.136 | 0.061 | -0.255 | -0.017 | -2.238 | 0.025 | * |
| CostDistanceInlet × Wet3 | -0.034 | 0.010 | -0.054 | -0.014 | -3.283 | 0.001 | * |
| CostDistanceInlet × Dry3 | 0.010 | 0.010 | -0.010 | 0.030 | 1.007 | 0.314 |  |
| *Random effect* |  |  |  |  |  |  |  |
| Intercept (Year × Month) | 0.487 |  |  |  |  |  |  |
|  |  |  |  |  |  |  |  |
| *Model 5* |  |  |  |  |  |  |  |
| *Fixed effects* |  |  |  |  |  |  |  |
| Intercept | -3.374 | 0.292 | -3.946 | -2.801 | -11.556 | 0.000 | * |
| NOAA_DEM | 0.019 | 0.102 | -0.182 | 0.219 | 0.183 | 0.855 |  |
| CostDistanceInlet | 0.004 | 0.013 | -0.021 | 0.029 | 0.308 | 0.758 |  |
| Wet6 | 0.334 | 0.129 | 0.082 | 0.586 | 2.600 | 0.009 | * |
| Dry6 | -0.181 | 0.148 | -0.471 | 0.109 | -1.223 | 0.221 |  |
| Seagrass400 | 2.345 | 0.354 | 1.652 | 3.039 | 6.629 | 0.000 | * |
| Saltmarsh400 | -0.192 | 0.330 | -0.839 | 0.455 | -0.583 | 0.560 |  |
| Oysters400 | -1.220 | 0.874 | -2.932 | 0.492 | -1.396 | 0.163 |  |
| HabitatRichness | 0.318 | 0.103 | 0.115 | 0.521 | 3.076 | 0.002 | * |
| Latitude | -0.139 | 0.061 | -0.259 | -0.019 | -2.279 | 0.023 | * |
| CostDistanceInlet × Wet6 | -0.034 | 0.010 | -0.053 | -0.014 | -3.317 | 0.001 | * |
| CostDistanceInlet × Dry6 | 0.005 | 0.010 | -0.014 | 0.024 | 0.515 | 0.606 |  |
| *Random effect* |  |  |  |  |  |  |  |
| Intercept (Year × Month) | 0.498 |  |  |  |  |  |  |
