# Supplementary material for: The influence of freshwater inflow and seascape context on occurrence of juvenile spotted seatrout Cynoscion nebulosus across a temperate estuary
Source: PLoS One. 2023 Nov 28;18(11):e0294178. doi: 10.1371/journal.pone.0294178 (PMC10684023; doi:10.1371/journal.pone.0294178)
Supplement: S3 Table — Parameter estimates, standard errors, lower and upper 95% confidence limits, and Wald z-scores (z) and p-values (p) from the confidence set of mixed effects logistic regression models relating seascape-scale, estuary-scale, and hydrologic variables to the probability of encountering 101–150 mm spotted seatrout. All values are on the logit (log-odds) scale, random effects are reported as standard deviations, and Imp denotes statistically important relationships based on an alpha level of 0.05. (DOCX) [file pone.0294178.s003.docx]

| **S3 Table.** **Models for 101** – **150 mm Spotted seatrout.** Parameter estimates, standard errors, lower and upper 95% confidence limits, and Wald z-scores (*z*) and p-values (*p*) from the confidence set of mixed effects logistic regression models relating seascape-scale, estuary-scale, and hydrologic variables to the probability of encountering 101 – 150 mm spotted seatrout. All values are on the logit (log-odds) scale, random effects are reported as standard deviations, and Imp denotes statistically important relationships based on an alpha level of 0.05. | | | | | | | |
| --- | --- | --- | --- | --- | --- | --- | --- |
| Parameter | Estimate | SE | Lower | Upper | *z* | *p* | Imp |
| *Model 17* |  |  |  |  |  |  |  |
| *Fixed effects* |  |  |  |  |  |  |  |
| Intercept | -3.856 | 0.298 | -4.440 | -3.273 | -12.953 | 0.000 | * |
| NOAA_DEM | -0.308 | 0.143 | -0.587 | -0.028 | -2.156 | 0.031 | * |
| CostDistanceInlet | 0.094 | 0.013 | 0.069 | 0.120 | 7.338 | 0.000 | * |
| Seagrass400 | 2.489 | 0.365 | 1.773 | 3.205 | 6.816 | 0.000 | * |
| Saltmarsh400 | 0.663 | 0.622 | -0.555 | 1.881 | 1.066 | 0.286 |  |
| Oysters400 | -1.470 | 0.759 | -2.958 | 0.018 | -1.936 | 0.053 |  |
| HabitatRichness | 0.459 | 0.093 | 0.277 | 0.641 | 4.941 | 0.000 | * |
| Longitude | -0.601 | 0.083 | -0.764 | -0.438 | -7.217 | 0.000 | * |
| *Random effect* |  |  |  |  |  |  |  |
| Intercept (Year × Month) | 0.901 |  |  |  |  |  |  |
|  |  |  |  |  |  |  |  |
| *Model 5* |  |  |  |  |  |  |  |
| *Fixed effects* |  |  |  |  |  |  |  |
| Intercept | -3.839 | 0.297 | -4.422 | -3.257 | -12.922 | 0.000 | * |
| NOAA_DEM | -0.291 | 0.143 | -0.572 | -0.010 | -2.031 | 0.042 | * |
| CostDistanceInlet | 0.094 | 0.013 | 0.069 | 0.119 | 7.265 | 0.000 | * |
| Seagrass400 | 2.487 | 0.365 | 1.772 | 3.203 | 6.812 | 0.000 | * |
| Saltmarsh400 | 0.686 | 0.622 | -0.532 | 1.905 | 1.104 | 0.270 |  |
| Oysters400 | -1.496 | 0.763 | -2.991 | -0.001 | -1.962 | 0.050 | * |
| HabitatRichness | 0.456 | 0.093 | 0.274 | 0.639 | 4.907 | 0.000 | * |
| Wet6 | -0.199 | 0.165 | -0.522 | 0.124 | -1.209 | 0.227 |  |
| Dry6 | -0.167 | 0.160 | -0.481 | 0.148 | -1.039 | 0.299 |  |
| Longitude | -0.600 | 0.083 | -0.763 | -0.437 | -7.200 | 0.000 | * |
| CostDistanceInlet × Wet6 | 0.008 | 0.012 | -0.015 | 0.032 | 0.712 | 0.476 |  |
| CostDistanceInlet × Dry6 | 0.025 | 0.012 | 0.002 | 0.048 | 2.135 | 0.033 | * |
| *Random effect* |  |  |  |  |  |  |  |
| Intercept (Year × Month) | 0.884 |  |  |  |  |  |  |
|  |  |  |  |  |  |  |  |
| *Model 14* |  |  |  |  |  |  |  |
| *Fixed effects* |  |  |  |  |  |  |  |
| Intercept | -3.845 | 0.297 | -4.427 | -3.262 | -12.931 | 0.000 | * |
| NOAA_DEM | -0.302 | 0.143 | -0.582 | -0.022 | -2.114 | 0.035 | * |
| CostDistanceInlet | 0.094 | 0.013 | 0.069 | 0.119 | 7.328 | 0.000 | * |
| Seagrass400 | 2.476 | 0.365 | 1.761 | 3.192 | 6.787 | 0.000 | * |
| Saltmarsh400 | 0.676 | 0.621 | -0.541 | 1.894 | 1.089 | 0.276 |  |
| Oysters400 | -1.473 | 0.761 | -2.965 | 0.019 | -1.935 | 0.053 |  |
| HabitatRichness | 0.457 | 0.093 | 0.275 | 0.639 | 4.917 | 0.000 | * |
| Wet6 | -0.119 | 0.120 | -0.354 | 0.116 | -0.996 | 0.319 |  |
| Dry6 | 0.062 | 0.116 | -0.166 | 0.290 | 0.534 | 0.594 |  |
| Longitude | -0.597 | 0.083 | -0.761 | -0.434 | -7.177 | 0.000 | * |
| *Random effect* |  |  |  |  |  |  |  |
| Intercept (Year × Month) | 0.887 |  |  |  |  |  |  |
|  |  |  |  |  |  |  |  |
| *Model 16* |  |  |  |  |  |  |  |
| *Fixed effects* |  |  |  |  |  |  |  |
| Intercept | -3.907 | 0.306 | -4.506 | -3.307 | -12.770 | 0.000 | * |
| NOAA_DEM | -0.312 | 0.143 | -0.591 | -0.032 | -2.186 | 0.029 | * |
| CostDistanceInlet | 0.094 | 0.013 | 0.069 | 0.120 | 7.336 | 0.000 | * |
| Seagrass400 | 2.501 | 0.365 | 1.785 | 3.218 | 6.844 | 0.000 | * |
| Saltmarsh400 | 0.674 | 0.622 | -0.544 | 1.893 | 1.085 | 0.278 |  |
| Oysters400 | -1.483 | 0.759 | -2.970 | 0.004 | -1.955 | 0.051 |  |
| HabitatRichness | 0.457 | 0.093 | 0.275 | 0.639 | 4.918 | 0.000 | * |
| Wet1 | 0.125 | 0.295 | -0.454 | 0.703 | 0.422 | 0.673 |  |
| Dry1 | 0.673 | 0.617 | -0.536 | 1.882 | 1.092 | 0.275 |  |
| Longitude | -0.602 | 0.083 | -0.766 | -0.439 | -7.231 | 0.000 | * |
| *Random effect* |  |  |  |  |  |  |  |
| Intercept (Year × Month) | 0.895 |  |  |  |  |  |  |
|  |  |  |  |  |  |  |  |
| *Model 15* |  |  |  |  |  |  |  |
| *Fixed effects* |  |  |  |  |  |  |  |
| Intercept | -3.858 | 0.298 | -4.442 | -3.274 | -12.947 | 0.000 | * |
| NOAA_DEM | -0.308 | 0.143 | -0.588 | -0.028 | -2.156 | 0.031 | * |
| CostDistanceInlet | 0.094 | 0.013 | 0.069 | 0.120 | 7.340 | 0.000 | * |
| Seagrass400 | 2.490 | 0.365 | 1.774 | 3.206 | 6.813 | 0.000 | * |
| Saltmarsh400 | 0.662 | 0.622 | -0.556 | 1.881 | 1.065 | 0.287 |  |
| Oysters400 | -1.474 | 0.760 | -2.963 | 0.014 | -1.941 | 0.052 |  |
| HabitatRichness | 0.459 | 0.093 | 0.277 | 0.641 | 4.941 | 0.000 | * |
| Wet3 | 0.003 | 0.116 | -0.225 | 0.230 | 0.022 | 0.983 |  |
| Dry3 | 0.029 | 0.114 | -0.195 | 0.253 | 0.252 | 0.801 |  |
| Longitude | -0.601 | 0.083 | -0.764 | -0.438 | -7.213 | 0.000 | * |
| *Random effect* |  |  |  |  |  |  |  |
| Intercept (Year × Month) | 0.902 |  |  |  |  |  |  |
|  |  |  |  |  |  |  |  |
| *Model 13* |  |  |  |  |  |  |  |
| *Fixed effects* |  |  |  |  |  |  |  |
| Intercept | -3.857 | 0.298 | -4.440 | -3.273 | -12.953 | 0.000 | * |
| NOAA_DEM | -0.308 | 0.143 | -0.588 | -0.028 | -2.156 | 0.031 | * |
| CostDistanceInlet | 0.094 | 0.013 | 0.069 | 0.120 | 7.338 | 0.000 | * |
| Seagrass400 | 2.489 | 0.365 | 1.773 | 3.205 | 6.815 | 0.000 | * |
| Saltmarsh400 | 0.663 | 0.622 | -0.556 | 1.881 | 1.066 | 0.286 |  |
| Oysters400 | -1.470 | 0.759 | -2.958 | 0.018 | -1.936 | 0.053 |  |
| HabitatRichness | 0.459 | 0.093 | 0.277 | 0.641 | 4.941 | 0.000 | * |
| Wet12 | -0.001 | 0.130 | -0.256 | 0.255 | -0.006 | 0.995 |  |
| Dry12 | -0.004 | 0.131 | -0.261 | 0.253 | -0.032 | 0.975 |  |
| Longitude | -0.601 | 0.083 | -0.764 | -0.438 | -7.216 | 0.000 | * |
| *Random effect* |  |  |  |  |  |  |  |
| Intercept (Year × Month) | 0.901 |  |  |  |  |  |  |
