# Supplementary material for: The influence of freshwater inflow and seascape context on occurrence of juvenile spotted seatrout Cynoscion nebulosus across a temperate estuary
Source: PLoS One. 2023 Nov 28;18(11):e0294178. doi: 10.1371/journal.pone.0294178 (PMC10684023; doi:10.1371/journal.pone.0294178)
Supplement: S4 Table — Parameter estimates, standard errors, lower and upper 95% confidence limits, and Wald z-scores (z) and p-values (p) from the confidence set of mixed effects logistic regression models relating seascape-scale, estuary-scale, and hydrologic variables to the probability of encountering 151–200 mm spotted seatrout. All values are on the logit (log-odds) scale, random effects are reported as standard deviations, and Imp denotes statistically important relationships based on an alpha level of 0.05. (DOCX) [file pone.0294178.s004.docx]

| **S4 Table.** **Models for 151** – **200 mm Spotted seatrout.** Parameter estimates, standard errors, lower and upper 95% confidence limits, and Wald z-scores (*z*) and p-values (*p*) from the confidence set of mixed effects logistic regression models relating seascape-scale, estuary-scale, and hydrologic variables to the probability of encountering 151 – 200 mm spotted seatrout. All values are on the logit (log-odds) scale, random effects are reported as standard deviations, and Imp denotes statistically important relationships based on an alpha level of 0.05. | | | | | | | |
| --- | --- | --- | --- | --- | --- | --- | --- |
| Parameter | Estimate | SE | Lower | Upper | *z* | *p* | Imp |
| *Model 17* |  |  |  |  |  |  |  |
| *Fixed effects* |  |  |  |  |  |  |  |
| Intercept | -3.724 | 0.306 | -4.323 | -3.124 | -12.180 | 0.000 | * |
| NOAA_DEM | -0.380 | 0.142 | -0.659 | -0.102 | -2.677 | 0.007 | * |
| CostDistanceInlet | 0.068 | 0.014 | 0.041 | 0.095 | 4.964 | 0.000 | * |
| Seagrass400 | 2.120 | 0.383 | 1.371 | 2.870 | 5.543 | 0.000 | * |
| Saltmarsh400 | 0.821 | 0.700 | -0.551 | 2.193 | 1.172 | 0.241 |  |
| Oysters400 | -0.582 | 0.772 | -2.095 | 0.931 | -0.753 | 0.451 |  |
| HabitatRichness | 0.291 | 0.100 | 0.096 | 0.487 | 2.923 | 0.003 | * |
| Longitude | -0.321 | 0.087 | -0.491 | -0.152 | -3.711 | 0.000 | * |
| *Random effect* |  |  |  |  |  |  |  |
| Intercept (Year × Month) | 0.826 |  |  |  |  |  |  |
|  |  |  |  |  |  |  |  |
| *Model 16* |  |  |  |  |  |  |  |
| *Fixed effects* |  |  |  |  |  |  |  |
| Intercept | -3.657 | 0.311 | -4.266 | -3.048 | -11.775 | 0.000 | * |
| NOAA_DEM | -0.383 | 0.142 | -0.661 | -0.104 | -2.692 | 0.007 | * |
| CostDistanceInlet | 0.068 | 0.014 | 0.041 | 0.094 | 4.949 | 0.000 | * |
| Seagrass400 | 2.114 | 0.382 | 1.364 | 2.863 | 5.527 | 0.000 | * |
| Saltmarsh400 | 0.837 | 0.699 | -0.533 | 2.207 | 1.197 | 0.231 |  |
| Oysters400 | -0.617 | 0.770 | -2.127 | 0.892 | -0.801 | 0.423 |  |
| HabitatRichness | 0.291 | 0.100 | 0.096 | 0.487 | 2.927 | 0.003 | * |
| Wet1 | -0.416 | 0.300 | -1.003 | 0.171 | -1.389 | 0.165 |  |
| Dry1 | 0.375 | 0.598 | -0.796 | 1.547 | 0.628 | 0.530 |  |
| Longitude | -0.320 | 0.087 | -0.490 | -0.151 | -3.702 | 0.000 | * |
| *Random effect* |  |  |  |  |  |  |  |
| Intercept (Year × Month) | 0.809 |  |  |  |  |  |  |
|  |  |  |  |  |  |  |  |
| *Model 14* |  |  |  |  |  |  |  |
| *Fixed effects* |  |  |  |  |  |  |  |
| Intercept | -3.713 | 0.305 | -4.311 | -3.114 | -12.162 | 0.000 | * |
| NOAA_DEM | -0.374 | 0.142 | -0.653 | -0.096 | -2.633 | 0.008 | * |
| CostDistanceInlet | 0.068 | 0.014 | 0.041 | 0.094 | 4.955 | 0.000 | * |
| Seagrass400 | 2.105 | 0.382 | 1.357 | 2.854 | 5.510 | 0.000 | * |
| Saltmarsh400 | 0.836 | 0.699 | -0.534 | 2.207 | 1.196 | 0.232 |  |
| Oysters400 | -0.577 | 0.775 | -2.096 | 0.942 | -0.744 | 0.457 |  |
| HabitatRichness | 0.289 | 0.100 | 0.094 | 0.485 | 2.904 | 0.004 | * |
| Wet6 | -0.162 | 0.118 | -0.394 | 0.070 | -1.370 | 0.171 |  |
| Dry6 | 0.033 | 0.112 | -0.187 | 0.253 | 0.292 | 0.770 |  |
| Longitude | -0.318 | 0.086 | -0.487 | -0.148 | -3.675 | 0.000 | * |
| *Random effect* |  |  |  |  |  |  |  |
| Intercept (Year × Month) | 0.811 |  |  |  |  |  |  |
|  |  |  |  |  |  |  |  |
| *Model 15* |  |  |  |  |  |  |  |
| *Fixed effects* |  |  |  |  |  |  |  |
| Intercept | -3.713 | 0.305 | -4.312 | -3.115 | -12.165 | 0.000 | * |
| NOAA_DEM | -0.377 | 0.142 | -0.655 | -0.098 | -2.649 | 0.008 | * |
| CostDistanceInlet | 0.067 | 0.014 | 0.041 | 0.094 | 4.946 | 0.000 | * |
| Seagrass400 | 2.100 | 0.382 | 1.351 | 2.849 | 5.493 | 0.000 | * |
| Saltmarsh400 | 0.829 | 0.700 | -0.544 | 2.201 | 1.184 | 0.237 |  |
| Oysters400 | -0.570 | 0.773 | -2.084 | 0.944 | -0.738 | 0.461 |  |
| HabitatRichness | 0.291 | 0.100 | 0.096 | 0.486 | 2.926 | 0.003 | * |
| Wet3 | -0.135 | 0.116 | -0.362 | 0.091 | -1.169 | 0.242 |  |
| Dry3 | -0.110 | 0.116 | -0.337 | 0.118 | -0.945 | 0.344 |  |
| Longitude | -0.320 | 0.087 | -0.489 | -0.150 | -3.697 | 0.000 | * |
| *Random effect* |  |  |  |  |  |  |  |
| Intercept (Year × Month) | 0.812 |  |  |  |  |  |  |
|  |  |  |  |  |  |  |  |
| *Model 5* |  |  |  |  |  |  |  |
| *Fixed effects* |  |  |  |  |  |  |  |
| Intercept | -3.715 | 0.306 | -4.314 | -3.116 | -12.159 | 0.000 | * |
| NOAA_DEM | -0.367 | 0.143 | -0.647 | -0.087 | -2.572 | 0.010 | * |
| CostDistanceInlet | 0.068 | 0.014 | 0.041 | 0.095 | 4.962 | 0.000 | * |
| Wet6 | -0.276 | 0.168 | -0.606 | 0.054 | -1.638 | 0.101 |  |
| Dry6 | -0.126 | 0.160 | -0.439 | 0.187 | -0.791 | 0.429 |  |
| Seagrass400 | 2.110 | 0.382 | 1.361 | 2.859 | 5.522 | 0.000 | * |
| Saltmarsh400 | 0.859 | 0.699 | -0.512 | 2.229 | 1.228 | 0.219 |  |
| Oysters400 | -0.595 | 0.775 | -2.114 | 0.925 | -0.767 | 0.443 |  |
| HabitatRichness | 0.288 | 0.100 | 0.092 | 0.483 | 2.885 | 0.004 | * |
| Longitude | -0.318 | 0.086 | -0.487 | -0.148 | -3.677 | 0.000 | * |
| CostDistanceInlet × Wet6 | 0.012 | 0.013 | -0.013 | 0.037 | 0.965 | 0.335 |  |
| CostDistanceInlet × Dry6 | 0.018 | 0.012 | -0.006 | 0.042 | 1.442 | 0.149 |  |
| *Random effect* |  |  |  |  |  |  |  |
| Intercept (Year × Month) | 0.809 |  |  |  |  |  |  |
|  |  |  |  |  |  |  |  |
| *Model 13* |  |  |  |  |  |  |  |
| *Fixed effects* |  |  |  |  |  |  |  |
| Intercept | -3.725 | 0.306 | -4.325 | -3.126 | -12.179 | 0.000 | * |
| NOAA_DEM | -0.379 | 0.142 | -0.658 | -0.101 | -2.669 | 0.008 | * |
| CostDistanceInlet | 0.068 | 0.014 | 0.041 | 0.095 | 4.971 | 0.000 | * |
| Seagrass400 | 2.120 | 0.383 | 1.370 | 2.869 | 5.541 | 0.000 | * |
| Saltmarsh400 | 0.825 | 0.700 | -0.547 | 2.196 | 1.178 | 0.239 |  |
| Oysters400 | -0.585 | 0.773 | -2.100 | 0.930 | -0.757 | 0.449 |  |
| HabitatRichness | 0.291 | 0.100 | 0.096 | 0.486 | 2.920 | 0.003 | * |
| Wet12 | -0.043 | 0.129 | -0.296 | 0.209 | -0.336 | 0.737 |  |
| Dry12 | 0.041 | 0.128 | -0.210 | 0.291 | 0.318 | 0.750 |  |
| Longitude | -0.320 | 0.087 | -0.490 | -0.151 | -3.700 | 0.000 | * |
| *Random effect* |  |  |  |  |  |  |  |
| Intercept (Year × Month) | 0.827 |  |  |  |  |  |  |
|  |  |  |  |  |  |  |  |
| *Model 8* |  |  |  |  |  |  |  |
| *Fixed effects* |  |  |  |  |  |  |  |
| Intercept | -3.728 | 0.306 | -4.328 | -3.129 | -12.187 | 0.000 | * |
| NOAA_DEM | -0.373 | 0.143 | -0.652 | -0.093 | -2.616 | 0.009 | * |
| Wet3 | -0.165 | 0.163 | -0.485 | 0.155 | -1.010 | 0.313 |  |
| Dry3 | -0.286 | 0.169 | -0.617 | 0.046 | -1.689 | 0.091 |  |
| CostDistanceInlet | 0.069 | 0.014 | 0.042 | 0.096 | 5.017 | 0.000 | * |
| Seagrass400 | 2.108 | 0.383 | 1.358 | 2.858 | 5.508 | 0.000 | * |
| Saltmarsh400 | 0.832 | 0.700 | -0.541 | 2.204 | 1.188 | 0.235 |  |
| Oysters400 | -0.591 | 0.774 | -2.107 | 0.926 | -0.764 | 0.445 |  |
| HabitatRichness | 0.291 | 0.100 | 0.096 | 0.486 | 2.924 | 0.003 | * |
| Longitude | -0.322 | 0.087 | -0.492 | -0.152 | -3.712 | 0.000 | * |
| CostDistanceInlet × Wet3 | 0.003 | 0.013 | -0.022 | 0.028 | 0.255 | 0.799 |  |
| CostDistanceInlet × Dry3 | 0.019 | 0.012 | -0.005 | 0.044 | 1.522 | 0.128 |  |
| *Random effect* |  |  |  |  |  |  |  |
| Intercept (Year × Month) | 0.812 |  |  |  |  |  |  |
|  |  |  |  |  |  |  |  |
| *Model 11* |  |  |  |  |  |  |  |
| *Fixed effects* |  |  |  |  |  |  |  |
| Intercept | -3.654 | 0.314 | -4.270 | -3.038 | -11.626 | 0.000 | * |
| NOAA_DEM | -0.381 | 0.142 | -0.660 | -0.102 | -2.681 | 0.007 | * |
| CostDistanceInlet | 0.067 | 0.015 | 0.038 | 0.096 | 4.547 | 0.000 | * |
| Wet | -0.327 | 0.416 | -1.141 | 0.488 | -0.786 | 0.432 |  |
| Dry | -0.139 | 0.853 | -1.810 | 1.533 | -0.163 | 0.871 |  |
| Seagrass400 | 2.114 | 0.382 | 1.364 | 2.863 | 5.528 | 0.000 | * |
| Saltmarsh400 | 0.831 | 0.700 | -0.541 | 2.202 | 1.187 | 0.235 |  |
| Oysters400 | -0.654 | 0.772 | -2.166 | 0.858 | -0.847 | 0.397 |  |
| HabitatRichness | 0.294 | 0.100 | 0.099 | 0.490 | 2.951 | 0.003 | * |
| Longitude | -0.321 | 0.087 | -0.491 | -0.151 | -3.709 | 0.000 | * |
| CostDistanceInlet × Wet | -0.010 | 0.034 | -0.077 | 0.056 | -0.308 | 0.758 |  |
| CostDistanceInlet × Dry | 0.053 | 0.060 | -0.065 | 0.171 | 0.878 | 0.380 |  |
| *Random effect* |  |  |  |  |  |  |  |
| Intercept (Year × Month) | 0.808 |  |  |  |  |  |  |
